# Use of Passive and Grab Sampling and High-Resolution Mass Spectrometry for Non-Targeted Analysis of Emerging Contaminants and Their Semi-Quantification in Water

**DOI:** 10.3390/molecules27103167

**Published:** 2022-05-16

**Authors:** Đorđe Tadić, Rayana Manasfi, Marine Bertrand, Andrés Sauvêtre, Serge Chiron

**Affiliations:** 1 Hydrosciences Montpellier, University Montpellier, CNRS, IRD, 34090 Montpellier, France; rayana.manasfi@umontpellier.fr (R.M.); serge.chiron@umontpellier.fr (S.C.); 2 Hydrosciences Montpellier, University Montpellier, IMT Mines Ales, CNRS, IRD, 30100 Ales, France; marine.bertrand@mines-ales.fr (M.B.); andre.sauvetre@mines-ales.fr (A.S.)

**Keywords:** non-target screening, passive sampling, contaminants of emerging concern, transformation products, wastewater, river water, semi-quantification

## Abstract

Different groups of organic micropollutants including pharmaceuticals and pesticides have emerged in the environment in the last years, resulting in a rise in environmental and human health risks. In order to face up and evaluate these risks, there is an increasing need to assess their occurrence in the environment. Therefore, many studies in the past couple of decades were focused on the improvements in organic micropollutants’ extraction efficiency from the different environmental matrices, as well as their mass spectrometry detection parameters and acquisition modes. This paper presents different sampling methodologies and high-resolution mass spectrometry-based non-target screening workflows for the identification of pharmaceuticals, pesticides, and their transformation products in different kinds of water (domestic wastewater and river water). Identification confidence was increased including retention time prediction in the workflow. The applied methodology, using a passive sampling technique, allowed for the identification of 85 and 47 contaminants in the wastewater effluent and river water, respectively. Finally, contaminants’ prioritization was performed through semi-quantification in grab samples as a fundamental step for monitoring schemes.

## 1. Introduction

Pharmaceuticals are continuously introduced into the environment, and in recent years their usage increased, especially in response to the pandemic caused by the outbreak of the coronavirus (SARS-CoV-2) [1,2]. Many studies, published in the last decade, provide information on the occurrence and fate of pharmaceuticals in the aquatic environment, e.g., surface waters [3] and wastewater effluents (WWE) [2]. These compounds could be resistant to environmental degradation and are considered as one of the major categories of contaminants of emerging concern (CEC) [2,4]. In addition to pharmaceuticals, pesticides are of major concern as well, as compounds that are stable over time and harmful not only for human health but also can cause profound imbalances in the ecosystem [5,6].

Current monitoring of CEC in water bodies is mainly focused on the quantification of several compounds, which are selected based on the various prioritization schemes [7,8]. Although this approach has its advantages, it is rather limited since only a small subgroup of chemicals is revealed. Screening and quantification of a big number of analytes applying the conventional approach require analytical standards, which are costly and sometimes not even commercially available (e.g., for (bio)transformation products (TPs)). Another drawback of target screening is overlooking contaminant metabolites and TPs that can, in some cases, retain biological activity and/or be more abundant and harmful than their parent compounds [9,10]. In fact, the path from discharge to complete mineralization is rather long and complex, and it was demonstrated that incomplete mineralization of one parent compound yields several TPs [11]. Concern about their occurrence is gaining more and more scientific attention; for instance, O-desmethyl venlafaxine was already included in the Water Watch List under the Water Framework Directive (https://eur-lex.europa.eu/legal-content/EN/TXT/?uri=uriserv:OJ.L_.2020.257.01.0032.01.ENG&toc=OJ:L:2020:257:TOC, accessed on 1 February 2022). Hence, monitoring practitioners and policymakers should consider new techniques to achieve a more holistic water quality assessment against organic micropollutants and to reveal a bigger fraction of the entire chemical profile. In this way, other substances, which were not included in the target monitoring schemes and which are, for example, of local concern, can be considered [12,13]. For instance, Gago-Ferrero et al. demonstrated the application of high-resolution mass spectrometry (HRMS) with 24 h composite flow proportional samples in a successfully performed wide scope screening for more than 2000 compounds in wastewater [14].

Nowadays, we are witnessing a shift in the approach when it comes to contaminants’ monitoring in the environment, especially water. Qualitative data accompanied with semi-quantitative data can be more valuable than exact concentration for only a few compounds [12]. Analytical possibilities with new generation instrumentation, such as liquid or gas chromatography coupled with HRMS, are providing a possibility for new and comprehensive assessment, yet there is still space and need for optimization and harmonization of analytical procedures. As stated by Menger et al. [15], recent progress in non-target screening (NTS) analysis requires new challenges for quality control and quality assurance.

In this context, we evaluated the advantages and disadvantages of grab and passive sampling, and prioritization of contaminants based on the identification confidence and their concentrations. The applied methodology includes liquid chromatography coupled with HRMS applied for the identification of CEC in two different types of water, i.e., municipal wastewater effluent and river water. Identification was based on mass accuracy, isotopic pattern, fragmentation pattern in the deconvoluted MS^2^, and retention time (RT) prediction. Finally, detected compounds were semi-quantified using the in-silico ionization efficiency prediction. Therefore, we are suggesting highly confident rapid screening of CEC and their TPs by NTS and their semi-quantification as a step forward in the management of water pollution and as a fundament for establishing new monitoring schemes.

## 2. Results

### 2.1. Compound Identification

**Figure 1:**
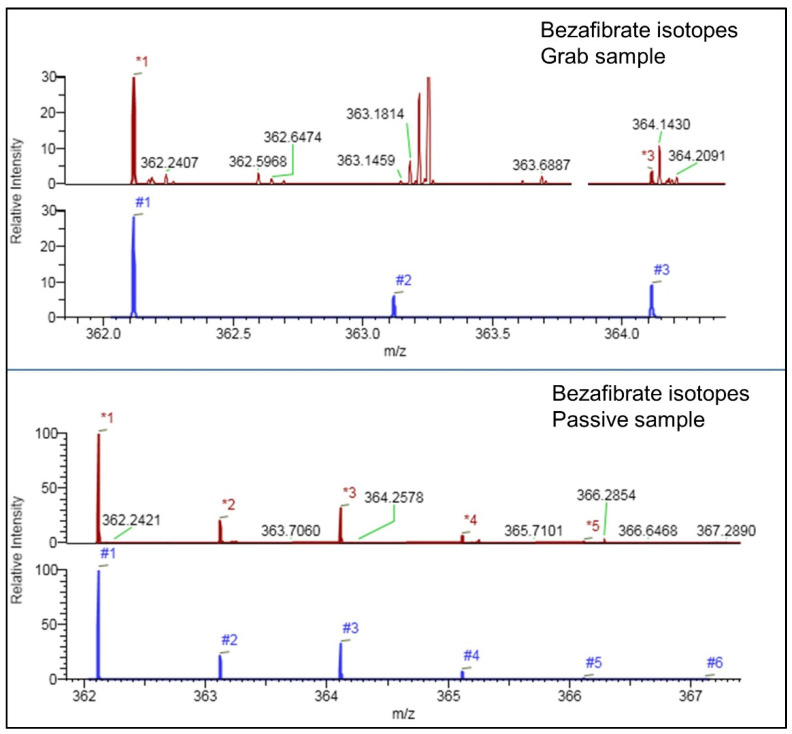
Detected isotopes of bezafibrate in grab and passive samples. Color indicates spectra type (red: experimental spectra; blue: theoretical spectra).

In total, 118 compounds were detected in WWE (n = 85) and river samples (n = 47) using HRMS of which 14 were common. Seven pesticides and 6 pharmaceuticals were confirmed with analytical standard or deuterated standard, reaching level 1 of identification, whereas the rest were identified by matching their DDA-MS^2^ spectra with the spectral libraries, hence reaching level 2a according to Schymanski et al. [16]. The difference between grab and passive sampling in terms of the number of identified compounds in the case of the WWE showed that five compounds (amisulpride N-oxide, clarithromycin, levorphanol, ofloxacin, and propranolol) were not detected in grab samples in spite of the potential relevance of antibiotics. Although these five compounds count for a relatively low percentage (6% of the total), their occurrence would remain undetected without applying passive samplers. One more advantage of passive samples over grab samples was observed during the identification process. Namely, the ion abundance in the mass spectra was higher, and this was especially important for the identification of the contaminants, as they provided a better quality of MS^2^ spectra. In general, only a small number of compounds would not pass confirmation due to poor isotopic pattern match. For example, for bezafibrate, four out of four isotopes were present in the passive sample, while only the main mass of bezafibrate was detected in grab samples, and none of the other isotopes (as presented in Figure 1); hence, this compound was filtered as identified but not confirmed, while in the passive sample, bezafibrate was identified and confirmed. A similar situation was observed for anabasine and metformin where the isotopic pattern match was lower than 80% in grab samples. 

**Figure 2:**
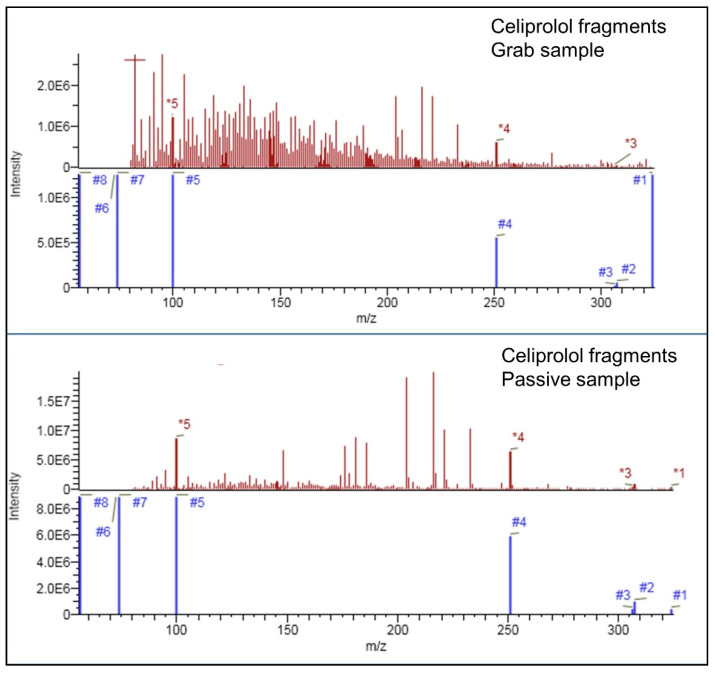
Detected fragments of celiprolol in data-independent acquisition mode in grab and passive samples. Color indicates spectra type (red: experimental spectra; blue: theoretical spectra).

As mentioned, all compounds were identified based on the occurrence of at least two fragments in their MS^2^ spectra, and in this regard, to fulfill this minimal requirement, there were no differences between grab and passive samples. However, a higher number of fragments was observed in passive samples. In addition, the difference in the intensity was observed too, showing their prevalence over the background matrix (see Figure 2). Due to the aforementioned and the nature of non-target screening, there is a possibility of false positives and false negatives. The authors support the application of passive samplers for the screening of CEC in WWE because passive samplers provided results with higher confidence. More specifically, the higher number of detected fragments and better isotopic pattern match due to higher concentrations of CEC in extracts obtained from passive samplers.

On the other hand, results obtained from the river water also showed a clear advantage of passive sampling over the grab sampling, which is also reflected in the number of identified compounds, which is in accordance with findings by Mathon et al. [17]. Data presented in the Supplementary excel file (“Summary”) show a difference not just between grab and passive samples, but also between grab samples themselves when sampled on a different date. Namely, 47 compounds were detected in passive samples while in the grab samples 24 and 17 compounds were detected, on the day of the placement and after 14 days of placement, respectively. Moreover, two compounds (trimethoprim and ethylmorphine) were identified in the grab sample and not in POCIS, which was not the case when WWE was analyzed. This difference in the qualitative results can be attributed to the fluctuation in river flow and in CEC concentration. Consequently, more consistent qualitative data obtained in WWE can be attributed to the fact that the sampling was conducted in the maturation pond which has a hydraulic RT of several days enabling homogenization; hence, fluctuations are less evident. 

Target analysis is indeed a valuable approach due to the unequivocal identification of individual compounds at very low trace levels, but it becomes cumbersome and expensive when the screening of a large number of analytes is required [18]. On the contrary, as demonstrated in this study, NTS is capable of identifying a higher number of contaminants with level 2a confidence in comparison with level 1 confidence which is labor-intensive, especially for the relatively big number of potential contaminants in a sample. However, unless we reach level 1, there is always a concern related to the confidence of the results. In the presented workflow, MS^2^ spectra of all compounds, obtained by data-dependent acquisition, matched their spectra from online databases. In this way, fragmentation patterns and so-called characteristic fragments were manually compared to reduce the possibility of false-positive outputs. However, the investigation of fragmentation patterns and characteristic fragments of individual compounds is time-consuming and should be automatized. Another strategy, commonly used to reduce the chance of false-positive results, is RT prediction, which is discussed in the following section.

### 2.2. Retention Time Prediction

**Figure 3:**
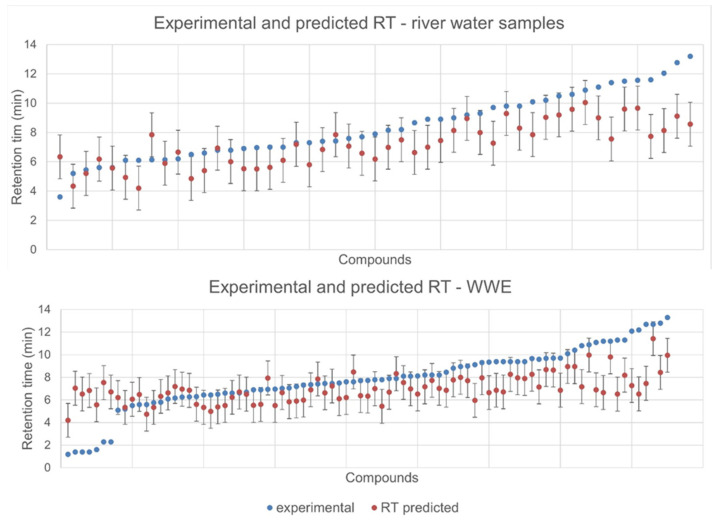
Experimental and predicted RT in WWE and river water. Acceptable error (1.5 min) of the predicted RT is depicted as error bar.

It is important to say, as stated by Bade et al. [19], that the application of RT prediction is not to replace the use of reference standards, but to help to gain more confidence in the obtained data along with MS^2^ data. As an important parameter that can refine obtained results, RT prediction is gaining attention in the scientific community; hence, simple algorithms based on the log Kow value and more complex ones based on the quantitative structure-retention relationship were developed [19,20]. The acceptable error for predicted RT can be estimated as 12% of the total run time, or 12% of the maximum experimental RT used in the training set during model development [20], whereas the window of 2 min was used in another study [19]. In this study, we applied the aforementioned estimation based on the 12% of maximum RT used in the training set, as this error window (1.5 min) is the narrowest. Figure 3 shows experimental and predicted RT with allowed error bars. As it can be seen, both data sets’ (WWE and river samples) compounds eluted at the beginning of the chromatographic run and at the end did not fit the applied error window. Nonetheless, 73% and 71% of all compounds in river and WWE, respectively, fit the model within the acceptable error window.

After the 11th min and at the beginning of the chromatographic run, we observed a difference bigger than the accepted error window (Figure 3); consequently, these compounds were marked as less confident results, but were not discarded completely (Supplementary excel file “Summary”). RT prediction can help us during the prioritization of identified compounds especially when accompanied with concentration data; in this way, we can select relevant contaminants with high identification confidence. 

RT prediction is generally used for the discrimination of possible false-positive results, especially in cases where several peaks are detected for one compound in an EIC (extracted ion chromatogram). However, there are other possible explanations and scenarios that have to be considered before a priori rejection of signals that do not fit our prediction model. Hypothetically speaking, if the DDA-MS^2^, including fragmentation pattern and characteristic fragments of a certain compound, matches the online library but the identified compound does not match RT prediction, it can originate from another molecule from which it was released during the ionization. Here, the authors would like to stress the possibility of “hidden” parent compounds revealed by in-source fragmentation. This might happen with biotransformation products, especially phase II metabolites, where added moiety “disappeared” as neutral loss, and here we detected the remaining part of the metabolite. Some biotransformation products, such as sugar or glutathione conjugates, are by default more polar than the parent compound; hence, we can expect their elution at the beginning of the chromatographic run in the given conditions. It was confirmed that weak bonds in a metabolite can be broken during the ionization process, even using the softest ionization method [21,22], while the higher temperatures can increase in source fragmentation, yielding the even greater response of fragments than the parent compounds [23].

Due to the aforementioned, we should not discard these results, but rather keep in mind that they might originate from a metabolite, especially in environmental samples. This can be further investigated; for example, neutral loss detection can be applied for the identification of possible metabolites from which the detected compound occurred, whereas multi-stage mass spectrometry (MS^n^) can further aid in structure elucidation. 

### 2.3. Contamination Profile

**Figure 4:**
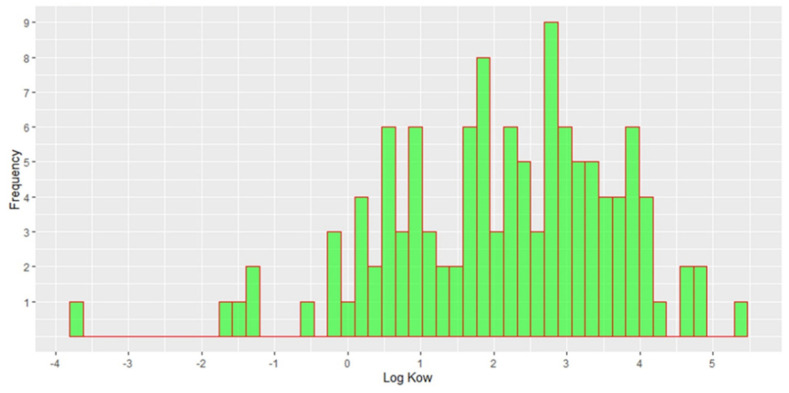
Histogram of log Kow values of all detected compounds.

Log Kow values of detected compounds ranged from −3.8 to 5.3 with mean and median values of 2.1 and 2.2, respectively, and their distribution is presented in Figure 4. As it can be seen, if we exclude two extreme values, we can observe that log Kow values ranged from −2 to 5. The choice of sorbents may affect the pre-selection of compounds based on the affinity of the analytes [24], which is clearly demonstrated by the log Kow range. For the sake of comprehensiveness of NTS, for the more polar compounds and more polar metabolites and TPs with log Kow < −2, other sorbents than HLB (e.g., multilayer cartridges) and other chromatographic conditions (e.g., HILIC column) are needed. 

For compounds detected in river water samples, log Kow values ranged from −0.25 to 4.8, with mean and median values of 2.2, while in WWE, 70% of all compounds detected have log Kow less than 3. This is an important threshold because compounds with log Kow values less than 3 do not adsorb greatly to the particles; hence, their occurrence in the WWE is expected. On the other hand, compounds with log Kow values higher than 3 have medium or high adsorption on the particles, which increases the hydraulic retention time of these compounds during the treatment and consequently their better removal during the treatment [25]. However, we detected 25 compounds in the WWE with log Kow values higher than 3. As stated by Pomiès et al.’s [26] prediction, the removal in the WWTP is rather difficult due to the number of mechanisms. Therefore, a comprehensive and robust analytical technique is necessary when it comes to the screening of the WWE. Moreover, compounds with higher log Kow values than 3 should not be a priory excluded from the target lists. 

**Figure 5:**
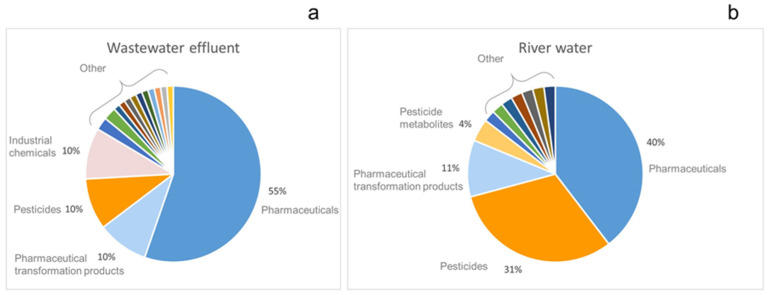
Distribution pattern of detected compounds according to their group in (**a**) wastewater effluent and (**b**) river water.

Among 85 compounds that were detected in the municipal WWE, 65% were pharmaceuticals and their TPs (Figure 5a), followed by pesticides and industrial chemicals both counting for 10%. The rest of identified compounds belonged to the group of steroids, rodenticides, illicit drugs, insect repellents, neurotransmitters, etc. (Supplementary excel file “Summary”). Among pharmaceuticals, the predominant groups were: antibacterial (n = 6), cardiovascular medications (n = 6), sedative (n = 5), non-steroidal anti-inflammatory drugs (NSAIDs) (n = 5), pain killers (n = 4), antiepileptic (n = 3), antifungal (n = 3), antihistaminic (n = 2), beta-blocker (n = 2), lipid-lowering (n = 2), neuropathic pain relief (n = 2), whilst for the following, only one compound per group was detected: anticholinergic, antidepressant, cough suppressant, antidiabetic, neuromuscular weakness, scabicidal agent, and stimulant (antitobacco). The observed contaminant profile shows the occurrence of pharmaceuticals used mainly to treat chronic diseases, and these groups of pharmaceuticals are commonly detected in water [1]. Obtained results are in accordance with previously published articles where a comprehensive screening of contaminants in wastewater was conducted. Namely, 43 compounds identified in this study were also present in the wastewater from Athens [14]. 

In addition to parent compounds, 9 pharmaceutical TPs were detected, i.e., 10-hydroxycarbamazepine, carbamazepine epoxide, 2-amino-1H-benzimidazole, amisulpride N-oxide, gabapentin lactam, N-desmethyl mephenytoin (nirvanol), N-desmethyl clobazam, N-desmethyl venlafaxine, and O-desmethyl venlafaxine.

2-amino-1H-benzimidazole was reported as a TP of carbendazim [27], whereas its parent compound was not detected. Similarly, amisulpride N-oxide was detected, but not amisulpride, which leads us to the conclusion that some TPs are more persistent than their parent compounds. The same phenomenon was observed for nirvanol, a metabolite of mephenytoin used to control seizures [28]. One of the main concerns related to the occurrence of pharmaceutical TPs is their toxicity and biological activity, such as in the case of N-desmethyl clobazam, the main metabolite of clobazam, which shows a greater half-life than parent compounds and can retain pharmacologic activities [29]. Two metabolites of carbamazepine (10-hydroxycarbamazepine and carbamazepine epoxide) and venlafaxine (N-desmethyl venlafaxine and O-desmethyl venlafaxine) were detected alongside their parent compounds, raising a question of their possible synergistic effect as they appear in a cocktail in the WWE. 

One illicit drug, buphedrone, which belongs to the synthetic cathinone, was identified in the WWE, which is widely used since it is frequently detected in wastewaters across Europe [30]. Another synthetic cathinone, 3,4-dimethylmethcathinone (3,4-DMMC), and one cocaine metabolite (ecgonine) were detected. Regarding industrial compounds, the most abundant were plasticizers (n = 4), and one compound per group was detected which belongs to the anticorrosive, dye carrier, catalyst, and emulsifier family. Half of the detected pesticides were insecticides, whereas the remaining were herbicides and fungicides. The difference in the pest profile shows that insecticides are prevalent in the residential areas in comparison with river water where herbicides were prevalent due to the nearby agricultural areas, which will be discussed in the following lines. 

Results obtained from river water showed a bit different contamination profile in comparison with municipal WWE (Figure 5b). Namely, the majority of identified compounds were pharmaceuticals (40%) and pesticides (31%), followed by pharmaceutical metabolites (11%) and pesticides’ TPs (4%). The detected pharmaceuticals belong to the following groups of sedative and painkiller (n = 8), antipsychotic and antidepressant (n = 5), beta-blocker (n = 3), antibiotic (n = 1), cardiovascular, appetite suppressant, and antifungal. The detected pharmaceutical profile is somehow expected due to the presence of a psychiatric hospital, and its wastewater discharge close to the sampling point. The second-largest group of contaminants was pesticides, and among them were herbicides (n = 8) and fungicides (n = 7) and one insecticide. The occurrence of these pesticides can be explained by the presence of agricultural fields and the fact that applied pesticides can leach into the surface water. For instance, imidacloprid, propyzamide, metolachlor, boscalid, and simazine were also detected in other French rivers [17]. Eight pharmaceutical TPs were identified in the river water and these are metabolites of venlafaxine, amisulpride, tramadol, and lamotrigine. Additionally, two pesticide TPs were identified: desethyl sebuthylazine and desmethyl norflurazon. Two illicit drugs were detected (cathinone and its metabolite methcathinone) and 4′-Methyl-α-pyrrolidinobutiophenone (novel stimulant drug). None of these compounds were detected in the WW effluent. Norephedrine, which was used in human medicine, was excluded from the market, but this product can be used as an illegal drug. 

### 2.4. Quantitative and Semi-Quantitative Analysis

Semi-quantification based on the prediction of the ionization efficiency requires at least five compounds with known concentrations measured in the same analysis [31]. As the target analysis of pesticides did not provide enough data to conduct prediction of the ionization efficiency, concentrations of 9 deuterated compounds (which were added to samples before analysis) were used as well to fulfill the requirements. Semi-quantification of compounds using passive sampling was not attempted because this requires the knowledge of the sampling rate (Rs) which is specific to each compound and given conditions, and critical for calculating the ambient concentration. Results obtained from quantification and semi-quantification, in grab samples, for WWE and the river are presented in Table 1 and Table 2, respectively. Concentration values for WWE ranged from to 0.1 ng/L (sulpiride) to 3.1 × 10^5^ ng/L (butoxytriglycol). Two compounds, ibuprofen and butoxytriglycol, were detected above 1000 ng/L in all grab samples; other compounds at the same level are dibutyl phthalate, tributyl phosphate, flecanide, and lamotrigine, whereas the rest of the detected compounds were below 1000 ng/L. 

Results obtained by semi-quantification for river water are in accordance with the published data. Pharmaceuticals in environmental waters are typically present in low ng/L concentrations [32]. Similarly, González-Gaya et al. reported relatively low concentrations of CECs in river and estuarine water in Spain, e.g., caffeine (28 ng/L), metformin (37 ng/L), primidone (92 ng/L), and gabapentin (23 ng/L) [18]. In WWE, results are also mostly consistent with those of a comprehensive study, where 90 WW treatment plants were included, revealing the following compounds as the most abundant: sucralose, benzotriazoles, several organophosphate ester flame retardants, and pharmaceuticals carbamazepine, tramadol, telmisartan, venlafaxine, irbesartan, fluconazole, oxazepam, fexofenadine, diclofenac, citalopram, codeine, bisoprolol, esprosartan, and antibiotics trimethoprim, ciprofloxacine, sulfamethoxazole, and clindamycine in concentrations ranging from few hundreds of ng/L to few µg/L [33].

Several different approaches have been tested to overcome the lack of analytical standards and evaluate the most accurate ones for the semi-quantification [34]. Two strategies, based on the assumptions that TPs have the same response as the parent compounds and that the internal standard eluting closest to the compound of interest will have a similar response factor, appeared to be less reliable when comparing to the approach based on the prediction of the ionization efficiency of the compounds in the ESI source. Namely, the quantification error accounted for 2.1 times of a compound concentration when the ionization prediction was applied [34]. In fact, the nature of semi-quantification approaches and the generated quantification error can be the source of variability between the replicates, and consequently yielded concentration results with relatively high standards deviation values, as it can be seen in Table 1 and Table 2.

The presented approach can be widely used for water monitoring purposes. For instance, in comprehensive wastewater-based epidemiology, comprehensive screening can be conducted relatively quickly and obtain the first insight into the possible range of concentrations of compounds of interest. Similarly, it can be used during the first steps in the development of the contaminants’ monitoring schemes that aim at monitoring locally relevant contaminants. This is relevant since contamination profiles might differ, as it was presented in this study. For example, among identified compounds in this study, tributyl phosphate is estimated to be present in a concentration higher than its ChV value (chronic toxicity) for Mysid (which is 50 ng/L) and hence might be relevant. Comparison of detected concentrations with available Predicted No Effect Concentrations [3] showed that there is no ecological effect. Moreover, antimicrobial compounds were detected at concentrations that will not promote resistance selection [35]. Recently, NTS has been applied in the evaluation of the contaminants’ removal efficiency in the WWTP, providing more information on their performance. In this case, a comparison of the peak area of the compounds of interest before and after the treatment can give us a clue about the treatment efficiency (removal percentage). However, these estimations will inevitably generate errors due to the different concentration—peak area ratios. That is to say, the observed percentage difference of peak areas does not necessarily reflect the same difference in percentage in the concentration. Additionally, the comparison of peak areas in different sample types is less accurate due to the difference in the ionization suppression/enhancement caused by a specific matrix. One more advantage of the ionization prediction tool is that the aforementioned errors are minimized, and more accurate results can be obtained since it was demonstrated that different biological matrices (different types of cereals), hence different matrix effects, do not affect prediction error [31].

## 3. Materials and Methods

### 3.1. Sample Collection and Processing 

Seasonal (Polar Organic Chemical Integrative Sampler (POCIS) campaigns were deployed in the spring and summer of 2021. At each sampling point, one cage containing 3 POCIS discs was at least 30 cm vertically submerged; the cage was weighted down by a ballast and finally left in the water for 15 d. AttractSPEPOCIS HLB was purchased from Affinisep (Le Houlme, France) with approximately 230 mg of the solid adsorbent N-vinylpyrrolidone-divinylbenzene (Oasis HLB). The POCIS sampling area was 41 cm^2^. The 3 mL polypropylene cartridges used to recover POCIS receiving phases were purchased from Supelco (Bellefonte, PA, USA). 

Upon retrieval, POCIS discs were individually washed with distilled water, sealed in their original aluminum bag, transported to the lab in an icebox, and stored under −20 °C until extraction. Receiving phases of the POCIS were transferred into 3 mL SPE cartridges, spiked with 50 μL of a mixture of deuterated compounds (1 mg/L methanol), and then eluted with 8 mL of methanol. After concentration under a gentle stream of nitrogen, final extracts (reconstituted in 1 mL 10% methanol) were filtered with 0.2 µm of the PTFE filter and analyzed by LC-HRMS.

### 3.2. Target Analysis 

Grab water samples were collected in duplicates in 1 L amber glass bottles at the same point as the POCIS was deployed. Sampling was performed at the deployment and retrieval of POCIS, after 14 d in surface (river) water and after 7 and 14 d in wastewater. All samples were transported to the lab in an icebox, filtered through a 0.45 µm glass fiber filter, and stored at 4 °C until extraction within 24 h. 

For pesticides’ analysis, grab samples (500 mL) were filtered through GF/F filters to eliminate suspended matter, spiked with 50 μL of atrazine d5 (1 ng μL^−1^ acetone), and extracted by solid-phase extraction (SPE) using Oasis HLB cartridges (500 mg sorbent, 6 cc, Waters). Prior to extraction, the Oasis HLB cartridges were activated with 5 mL of acetonitrile under vacuum, followed by 5 mL of methanol and 5 mL of ultrapure water. Before elution, they were dried under vacuum for 1 h. Analytes were recovered by eluting the cartridges with 8 mL of acetonitrile at a flow rate of 1 mL min^−1^. Extracts were analyzed with an Alliance HPLC system (Waters Series 2695) using a reverse-phase Phenomenex Kinetex Polar C18 (100 mm × 4.6 mm I.D × 2.6 µm particle size) column and a security guard UHPLC Polar C18 4.6 mm ID (Phenomenex). The mobile phase was composed of Milli-Q and acetonitrile, both with 0.05% formic acid, at a constant flow rate of 0.4 mL/min. The gradient was programmed so the proportion of acetonitrile increased from 60 to 100% in 10 min followed by stabilization for 2 min, before returning to initial conditions. This system was coupled with a triple quadrupole mass spectrometer Micromass Quattro micro API (Waters) fitted with an ESI source operating in positive ion mode. Argon was used as collision gas. The multiple reaction monitoring mode was used for the ion specific acquisition. Analytical data treatment were done with MassLynx software from Waters. Its Quantlynx interface enables the quantification of the targeted substances. Analytical parameters are presented in Appendix A (Table A1). The linearity, LOQs and LODs, precision, and accuracy of the analytical methods were carefully validated as described in Branchet et al. [36].

### 3.3. Non-Target Analysis 

For non-target screening, a generic approach in the sample preparation of grab samples was applied. Namely, for extraction, solid-phase extraction (SPE) OASIS HLB cartridges were used (250 mg sorbent, 6 cc, from Waters). Cartridges were conditioned with 10 mL methanol followed by 10 mL water; then, the water sample (V = 250 mL) was loaded followed with 10 mL of water for rinsing. SPE cartridges were then set under vacuum until total dryness and eluted with 6 mL methanol. Obtained eluates were set under a gentle nitrogen flow for complete evaporation, and finally, residues were reconstituted with 1 mL of the LC-MS/MS mobile phase (10/90, methanol/water, *v*/*v*), filtered with 0.2 µm of the PTFE filter, and transferred to vials ready for injection.

Extracts were analyzed on a HPLC Accelera 600 pump coupled to a Q-Orbitrap HRMS mass spectrometer (Thermo Fischer Scientific, Les Ullis, France) equipped with a heated electrospray ionization probe (HESI) source for detection. Chromatographic separation was conducted using Waters XBridge BEH C18 (2.1 × 150 mm and 2.5 µm particle size) equipped with a pre-column. The chromatography assays involved a 10 μL injection volume, a 0.30 mL/min flow rate, and a binary gradient of water (A) and acetonitrile (B), both containing 0.1% formic acid, as follows: 10% B at 0–1 min, 90% B at 10–23 min, 10% B at 24–29 min. HESI parameters were as follows: 40 arbitrary units (AU) sheath gas; 15 AU auxiliary gas; 300 °C capillary temperature; 200 °C heater temperature, and the electrospray voltage was set at 3.0 kV for the positive and 2.5 kV for the negative ionization mode. The S-lens radio frequency (RF) level was set at 100 AU. Full scan data were acquired at a resolution of 70,000 full width at half maximum (FWHM) with an automatic gain control (AGC) of 10^6^, 250 ms of the maximum ion injection time, and scan range 100–1000 *m*/*z*. Moreover, for MS^2^, data-dependent acquisition (DDA) and data-independent acquisitions (DIA) were achieved at a resolution of 17,500, two absolute collision energies (20 eV and 40 eV) isolation window 1 *m*/*z*, AGC of 5 × 10^4^, 150 ms of the maximum ion injection time, and scan range 50–1000 *m*/*z*. 

Chromatograms obtained by DIA were screened for contaminants using two lists of emerging substances “S1 MASSBANK” (https://www.norman-network.com/?q=node/236, assessed on 1 February 2022) and the list of contaminants EFS_HRAM_Compound_Database provided by Thermo Fisher Scientific. Identification parameters were at least 2 fragments, and the parent compound detected with a mass error less than 5 ppm and at an intensity higher than 10^4^, while 80% for the isotopic match was a threshold for confirmation. After the first screening, all possible candidates were selected for the further DDA-MS^2^. Obtained spectra were matched against online available databases (i.e., mz cloud, MassBank, MoNA) for the final confirmation, reaching identification level 2a [16].

### 3.4. Retention Time Prediction, Semi-Quantification, Data Analysis 

RT was predicted using the software package Quantitative Structure Retention Relationships (QSRR) Automator [37]. The training data set consisted of the RT of 75 compounds, previously injected in the same system and presented in the Supplementary excel file. Semi-quantification was performed based on the in-silico prediction of the response of the compounds in ESI [31] which was provided by Quantem Analytics, Tartu, Estonia (www.quantem.co, accessed on 1 February 2022). Data were processed using Xcalibur (Thermo Fisher Scientific, Les Ullis, France) and TraceFinder (Thermo Fisher Scientific, Les Ullis, France). All analyses were conducted in triplicates. 

## 4. Conclusions

The presented methods demonstrated the advantage of the passive samplers over the grab samples for the qualitative analysis. When it comes to the NTS, level of confidence becomes a crucial factor. In this respect, passive samples yielded the MS^2^ spectrum which provided much more information, i.e., characteristic fragments and isotopes. It was shown that in cases of rivers or other water bodies where there is a relatively high flow, the advantage of passive samples is clearly demonstrated. Relying only on a grab sampling is leading to the overlooking and underestimation of the contamination state. Moreover, RT prediction can reduce false positives and increase the confidence of identified compounds. Finally, for the sake of comprehensiveness, semi-quantification was crucial for the prioritization of the compounds that should be more thoroughly monitored and proved to be a relevant tool for reducing the necessity of a priori purchasing of analytical standards.

## Figures and Tables

**Table 1:** Concentrations (and standard deviations) obtained by quantification and semi-quantification in WWE in grab sample.

Compound	Concentration (ng/L)			Compound	Concentration (ng/L)		
	Grab Day 0	Grab Day 7	Grab Day 14		Grab Day 0	Grab Day 7	Grab Day 14
1,3-Dicyclohexylurea	5 (±2.9)	2.6 (±0.9)	11.3 (±3.5)	Gemfibrozil	499 (±260)	57.9 (±14)	47.9 (±14.4)
10-Hydroxycarbazepine	90.9 (±6.7)	26.6 (±3.8)	41.1 (±1)	Ibuprofen	6.8 × 10^4^ (±6.3 × 10^4^)	2.7 × 10^3^ (±954)	1.7 × 10^3^ (±894)
2,2,6,6-Tetramethyl-4-piperidinol	83.8 (±50.6)	39.6 (±9.3)	152 (±37.9)	Imidacloprid *	16.5 (±1.2)	7.4 (±0.7)	11.1 (±2.5)
2-Amino-1H-benzimidazole	49.7 (±27.6)	31.5 (±12.8)	154.5 (±64.1)	Irbesartan	7.2 (±9.1)	11.9 (±5.8)	28.3 (±28.4)
2-Hydroxyestrone	95.9 (±24)	46.3 (±4.9)	79.6 (±0.4)	Ketoprofen	3.6 (±0.09)	3.3 (±2)	
3,4-Dimethylmethcathinone	21.2 (±7.8)	12 (±2.4)	80.4 (±17.2)	Lacosamide	2.4 (±0.1)	1.1 (±0)	1.2 (±0.2)
3-Acetylindole	1.5 (±0.1)	0.7 (±0.1)	0.7 (±0.2)	Lamotrigine	1464 (±39)	626 (±146)	1263 (±270)
Alminoprofen	2 (±0.7)	1.6 (±0.4)	6 (±3.2)	Laurolactam	27.3 (±2.8)	12.4 (±3.3)	49.1 (±5.6)
Alprenolol	2.8 (±2.7)	2.8 (±1.8)	25.1 (±16.7)	Lidocaine	13.9 (±6.1)	18.5 (±6.2)	55.9 (±33.1)
Aminocarb	47 (±0.1)	23.1 (±5.6)	42.4 (±5.9)	lorazepam	7.6 (±3.3)	1.4 (±0.3)	19.8 (±1.6)
Amisulpride N-oxide				Lormetazepam	8.3 (±2.8)	2.1 (±0.5)	10.5 (±1.7)
Anabasine	5 (±0.6)	2.1 (±0.9)	33.2 (±7)	Metformin	26.1 (±7.2)	8.6 (±3.1)	1.9 (±1)
Ancymidol	1.3 (±0.3)	1.5 (±0.5)	3.7 (±0.3)	Mexacarbate	2 (±0.3)	1.2 (±0.3)	14.8 (±0.6)
Antipyrine (phenazone)	2.1 (±1.3)	0.8 (±0.5)	11.1 (±1.3)	N-desmethyl mephenytoin	6 (±4)		2.8 (±0.3)
Azoxystrobin *	1.6 (±0.09)	0.9 (±0.4)	1.2 (±0.2)	N-desmethyl clobazam	168 (±76.7)	29.6 (±6.2)	552 (±49.8)
Benzotriazole	224 (±3.5)	166 (±33.2)	273 (±19)	N-desmethyl venlafaxine	17.2 (±10.5)	15 (±7.5)	60.4 (±31.5)
Bezafibrate	11.1 (±9.7)	11.8 (±4.5)	3.2 (±2.6)	Nordazepam	10.7 (±3.7)	6.7 (±2.5)	23.9 (±6.4)
Buphedrone	2.9 (±1.6)	4 (±1.9)	1.7 (±0.8)	O-Desmethyl venlafaxine	1.3 (±1.1)	0.3 (±0)	13.5 (±9.5)
Bupivacaine		0.4 (±0.2)	3 (±1.4)	Picaridin	5 (±2.1)	2.2 (±0.6)	6.8 (±1.5)
Butoxytriglycol	3.1 × 10^5^ (±1.0 × 10^5^)	7.2 × 10^4^ (±1.7 × 10^4^)	3.6 × 10^4^ (±1.8 × 10^4^)	Prazepam	7.3 (±3.6)	5.5 (±2.3)	14 (±5)
Caffeine	616 (±394)	38.9 (±6.1)	295 (±39.5)	Propiconazole	1.4 (±0.5)	1 (±0.4)	3 (±1.1)
Carbamazepine	104 (±9.1)	96.5 (±12)	119 (±0.7)	Pseudotropine	11.9 (±5.2)	10.6 (±4.8)	45.9 (±13.1)
Carbamazepine epoxide	11.6 (±1.1)	5 (±1.2)	10.3 (±0.3)	Pyridostigmine	127 (±5.5)	62.5 (±17.4)	55.5 (±2.9)
Celiprolol	24.4 (±28.5)	21.6 (±14.2)	292 (±273)	Pyroquilon	46.9 (±4.8)	14.6 (±3.5)	31.9 (±0.9)
Cetirizine	0.5 (±0.6)	0.4 (±0.4)	20.6 (±15.8)	Rosuvastatin	5.2 (±6.4)	1.3 (±0.5)	0.7 (±0.02)
Climbazole	20.1 (±3)	7.7 (±2.5)	12.9 (±2.3)	Serotonin	11.2 (±1.3)	4.9 (±1.5)	15.6 (±1)
Crotamiton	9.5 (±5)	1.1 (±0.3)	20.1 (±0.9)	Sotalol	5.8 (±7.1)	1.1 (±0.6)	13.3 (±14.5)
Cytisine	5.1 (±1.4)	3.7 (±2)	8.7 (±0.9)	Sulfamethoxazole	63 (±7)	29.5 (±6.3)	61.5 (±0.7)
Dextromethorphan			0.4 (±0.2)	Sulpiride		0.1 (±0.03)	0.5 (±0.3)
Diazinon	0.5 (±0.3)	0.3 (±0.1)	1.4 (±0.6)	Terbuthylazin*	0.2 (±0.09)	0.2 (±0.2)	3.9 (±0.4)
Dibutyl phthalate	3870 (±1142)	998 (±76.8)	5224 (±872)	Tramadol	7.7 (±5.5)	8 (±4.2)	41.3 (±24.3)
Diclofenac	1 (±0.04)			Tributyl phosphate	1164 (±151)	476 (±11.9)	4739 (±218)
Diuron *	3.6 (±0.7)	2.4 (±1)	1.9 (±0.9)	Triclosan	59.6 (±0.5)		
DMACA Reagent	0.4 (±0.05)	0.2 (±0)	0.7 (±0.08)	Triethyl phosphate	771 (±287)	190 (±17.1)	229 (±11.1)
Ecgonine	6.6 (±0.6)	4.8 (±1.4)	0.7 (±0.6)	Triisopropanolamine	3.8 (±2.7)	1.1 (±0.5)	11.4 (±8)
Estriol	186 (±13.1)	62 (±14.2)	105 (±6)	Trimethoprim	0.7 (±0.02)		2.5 (±1.3)
Fexofenadine	0.3 (±0.5)	0.5 (±0.4)	7.9 (±9.9)	Tris (2-butoxyethyl) phosphate	96.7 (±52.7)	60.8 (±28.3)	263 (±87.4)
Flecainide	614 (±520)	491 (±158)	1813 (±327)	Tyramine	99.6 (±21.3)	39.4 (±8.6)	68.1 (±2.7)
Fluconazole	33.2 (±9.9)	17.5 (±5.6)	37.4 (±6.7)	Valpromide	518 (±36.9)	122 (±8.7)	30.4 (±4)
Gabapentin	195 (±29.4)	68.1 (±11.5)	117 (±15.4)	Venlafaxine	5.3 (±4)	6.4 (±3.5)	41.6 (±19.3)
Gabapentin lactam	13.4 (±1.1)	9.8 (±3.9)	43.2 (±5.3)				

* quantified with analytical standard.

**Table 2:** Concentrations (and standard deviations) obtained by quantification and semi-quantification in river in grab samples.

Compounds	Concentration (ng/L)		Compounds	Concentration (ng/L)	
	Grab Day 0	Grab Day 14		Grab Day 0	Grab Day 14
Benzophenone	4 (±1.4)	15.8 (±0.6)	Trimethoprim		1.4 (±0.5)
Antipyrine/phenazone	2.4 (±0.2)	0.2 (±0.04)	Clobazam	0.1 (±0.03)	
Desethyl sebuthylazine	11.1 (±0.8)	3.2 (±0.1)	Ethylmorphine	18.2 (±2.2)	29.6 (±1.9)
Atraton	5.3 (±0.5)	2 (±0.03)	Benalaxyl	0.8 (±0.1)	
Metribuzin	51.6 (±0.8)		Tiapride	0.2 (±0.2)	0.1 (±0.02)
Prometon	8.8 (±0.8)	0.2 (±0.002)	Retrorsine	2.3 (±0.07)	
Picaridin	1.1 (±1.5)	7.4 (±2.3)	Amisulpride	2.4 (±0.1)	
Norfentanyl	65.9 (±66.7)	65.9 (±26.5)	Fluopyram	2.1 (±0.3)	
Lidocaine		0.09 (±0.01)	Azoxystrobin *	11.7 (±0.4)	
Prometryn	1.1 (±0.01)	0.07 (±0.04)	Flecainide		0.3 (±0.03)
Meperidine	0.05 (±0.009)	0.5 (±0.02)	Sulpiride	9.8 (±1.7)	17.8 (±1.5)
Lamotrigine	4 (±0.7)	167.2 (±1.6)	Tramadol	4 (±0.1)	3.6 (±0.01)
Napropamide	8.2 (±0.8)		Simazin*	5.7 (±0.07)	
Metolachlor *	4.2 (±0.3)				

* quantified with analytical standard.

## Data Availability

Not applicable.

## Supplementary Materials

The following supporting information can be downloaded at: https://www.mdpi.com/article/10.3390/molecules27103167/s1, Table S1: Summary.

## Sample Availability

Samples of the compounds are available from the authors.

## Appendix A

**Table A1:** Quality assurance and quality control of applied targeted analysis for pesticides in wastewater effluents.

Class	Name	RT	Quantification Trace	Recovery Rates in WW (in%)	CAS	Chemical Formula	Log Kow	Analytical LOD (µg/L)	Analytical LOQ (µg/L)	LOQ in Grab Water Sample (ng/L)	LOD in Water Sample (ng/L)
IS	Simazine D5	3.33	207.1 > 129	-	220621-41-0	C_7_H_7_D_5_ClN_5_	-	-	-	-	-
Herbicide	Acetochlor	7.12	224 > 224	142	34256-82-1	C_14_H_20_ClNO_2_	4.14	4.1	13.6	12.2	40.7
Herbicide	Alachlor	7.06	270.1 > 237.9	138	15972-60-8	C_14_H_20_ClNO_2_	2.97	10.0	33.2	29.9	99.7
Herbicide	Atrazine	3.94	216 > 174	112	1912-24-9	C_8_H_14_ClN_5_	2.50	1.9	6.4	5.8	19.3
Herbicide	Atrazine-desethyl (DEA)	2.72	188 > 146	116	6190-65-4	C_6_H_10_ClN_5_	1.51	4.6	15.5	13.9	46.5
Herbicide	Chlortoluron	3.51	213.1 > 72.1	129	15545-48-9	C_10_H_13_ClN_2_O	2.50	6.0	20.2	18.1	60.5
Herbicide	DCPMU	3.33	219 > 127.1	10	3567-62-2	C_8_H_8_Cl_2_N_2_O	2.94	5.4	17.9	16.1	53.6
Herbicide	DCPU	2.9	205 > 127	0	2327-02-8	C_7_H_6_Cl_2_N_2_O	2.35	4.1	13.5	12.2	40.6
Herbicide	Terbuthylazine desethyl (DET)	3.22	202.1 >146.1	107	30125-63-4	C_7_H_12_ClN_5_	2.30	1.4	4.6	4.2	13.9
Herbicide	Atrazine desisopropyl (DIA)	2.54	174 > 104	154	1007-28-9	C_5_H_8_ClN_5_	0.32	4.8	16.0	14.4	48.1
Herbicide	Diuron	3.83	233.1 > 72	125	330-54-1	C_9_H_10_Cl_2_N_2_O	2.87	3.7	12.5	11.2	37.4
Herbicide	Flazasulfuron	3.78	408.1 > 182.1	7	104040-78-0	C_13_H_12_F_3_N_5_O_5_S	−0.06	4.5	15.1	13.6	45.3
Herbicide	Isoproturon	3.67	207 > 72	124	34123-59-6	C_12_H_18_N_2_O	2.50	3.6	12.1	10.9	36.2
Herbicide	Linuron	5.21	249 > 160	122	330-55-2	C_9_H_10_Cl_2_N_2_O_2_	3.00	3.5	11.8	10.6	35.3
Herbicide	Metolachlor	6.92	252 > 252	148	51218-45-2	C_15_H_22_ClNO_2_	3.40	6.9	23.0	20.7	69.0
Herbicide	Oxadixyl	2.95	279.1 > 132.1	98	77732-09-3	C_14_H_18_N_2_O_4_	0.65	6.4	21.2	19.1	63.7
Herbicide	Propyzamide	6.06	256 > 190	133	23950-58-5	C_12_H_11_Cl_2_NO	3.30	4.5	14.9	13.4	44.7
Herbicide	Prosulfocarb	9.52	252 > 91	124	52888-80-9	C_14_H_21_NOS	4.48	2.8	9.3	8.4	27.9
Herbicide	Simazine	3.33	202 > 124	100	122-34-9	C_7_H_12_ClN_5_	2.30	2.4	7.9	7.1	23.6
Herbicide	Simazine hydroxy	1.91	184 > 114	37	2599-11-3	C_7_H_13_N_5_O	1.67	3.8	12.8	11.5	38.4
Herbicide	Terbutylazine	5.22	230.1 > 174	124	5915-41-3	C_9_H_16_ClN_5_	3.40	5.9	19.8	17.8	59.4
Herbicide	Terbutylazine hydroxy	1.93	212.2 > 156	0	66753-07-9	C_9_H_17_N_5_O	1.29	2.5	8.4	7.5	25.1
Fungicide	Azoxystrobin	4.86	404 > 344	121	131860-33-8	C_22_H_17_N_3_O_5_	2.50	3.9	13.0	11.7	39.0
Fungicide	Carbendazim	2.11	192 > 160	17	10605-21-7	C_9_H_9_N_3_O_2_	1.48	2.8	9.2	8.3	27.5
Fungicide	Dimethomorph	4.05	388 > 301	165	110488-70-5	C_21_H_22_ClNO_4_	2.63	4.3	14.4	13.0	43.3
Fungicide	Epoxiconazole	5.22	330.01 > 121.03	157	135319-73-2	C_17_H_13_ClFN_3_O	3.30	3.0	10.0	9.0	29.9
Fungicide	Metalaxyl	3.67	280 > 220	125	57837-19-1	C_15_H_21_NO_4_	1.65	8.8	29.3	26.4	88.0
Fungicide	Penconazole	6.66	284.2 > 159.1	145	66246-88-6	C_13_H_15_Cl_2_N_3_	3.72	4.2	14.1	12.7	42.4
Fungicide	Prochloraz	4.59	378.04 > 310.18	138	67747-09-5	C_15_H_16_Cl_3_N_3_O_2_	3.50	5.0	16.7	15.0	50.0
Fungicide	Pyrimethanil	4.32	200 > 107	97	53112-28-0	C_12_H_13_N_3_	2.84	2.9	9.5	8.6	28.6
Fungicide	Tebuconazole	5.74	308 > 70	152	107534-96-3	C_16_H_22_ClN_3_O	3.49	3.0	9.9	8.9	29.6
Fungicide	Tetraconazole	5.33	372 > 159	146	112281-77-3	C_13_H_11_Cl_2_F_4_N_3_O	3.60	5.0	16.7	15.0	50.0
Insecticide	Imidacloprid	2.62	256.1 > 209.1	95	138261-41-3	C_9_H_10_ClN_5_O_2_	0.57	4.1	13.6	12.2	40.7
